# Isolation and characterization of a *spotted leaf 32* mutant with early leaf senescence and enhanced defense response in rice

**DOI:** 10.1038/srep41846

**Published:** 2017-01-31

**Authors:** Liting Sun, Yihua Wang, Ling-long Liu, Chunming Wang, Ting Gan, Zhengyao Zhang, Yunlong Wang, Di Wang, Mei Niu, Wuhua Long, Xiaohui Li, Ming Zheng, Ling Jiang, Jianmin Wan

**Affiliations:** 1 State Key Laboratory for Crop Genetics and Germplasm Enhancement, Jiangsu Plant Gene Engineering Research Center, Nanjing Agricultural University, Nanjing 210095, China; 2 National Key Facility for Crop Gene Resources and Genetic Improvement, Institute of Crop Science, Chinese Academy of Agricultural Sciences, Beijing 100081, China

## Abstract

Leaf senescence is a complex biological process and defense responses play vital role for rice development, their molecular mechanisms, however, remain elusive in rice. We herein reported a rice mutant *spotted leaf 32 (spl32*) derived from a rice cultivar 9311 by radiation. The *spl32* plants displayed early leaf senescence, identified by disintegration of chloroplasts as cellular evidence, dramatically decreased contents of chlorophyll, up-regulation of superoxide dismutase enzyme activity and malondialdehyde, as physiological characteristic, and both up-regulation of senescence-induced *STAY GREEN* gene and senescence-associated transcription factors, and down-regulation of photosynthesis-associated genes, as molecular indicators. Positional cloning revealed that *SPL32* encodes a ferredoxin-dependent glutamate synthase (Fd-GOGAT). Compared to wild type, enzyme activity of GOGAT was significantly decreased, and free amino acid contents, particularly for glutamate and glutamine, were altered in *spl32* leaves. Moreover, the mutant was subjected to uncontrolled oxidative stress due to over-produced reactive oxygen species and damaged scavenging pathways, in accordance with decreased photorespiration rate. Besides, the mutant showed higher resistance to *Xanthomonas oryzae* pv. *Oryzae* than its wild type, coupled with up-regulation of four pathogenesis-related marker genes. Taken together, our results highlight Fd-GOGAT is associated with the regulation of leaf senescence and defense responses in rice.

Leaf senescence, as the final stage of leaf development, is an important biological process and affected by intrinsic factors, such as organ age and environment conditions (like light, temperature and plant growth regulators)^1^,^2^,^3^,^4^,^5^. Lesion mimic mutant, termed as spotted leaf (spl) in rice, is often associated with early leaf senescence. Hence it provides a tool for dissecting molecular mechanisms about leaf senescence. For example, rapid leaf senescence occurs in *rls1 (rapid leaf senescence 1*) mutant, and *RLS1* encodes an uncharacterized NB-ARM (nucleotide binding site-armadillo domain) protein regulating programed cell death (PCD) during leaf senescence^6^. *SPL28*, encoding a clathrin-associated adaptor protein complex 1, initiates leaf senescence through regulating vesicular trafficking^7^.

Besides displaying necrotic spots, lesion mimic mutants spontaneously activate plant defense response. Previous study reported defense response such as the resistance to bacterial blight inoculation was enhanced in 21 lesion mimic mutants isolated from IR64 rice mutant populations^8^, and similarly the resistance to rice blast fungus inoculation was increased in five lesion mimic mutants: *spl5-2, spl12, spl13, spl14*, and *spl15*^9^. Along with elevated resistance, expressions of some pathogenesis-related (PR) genes were induced in rice lesion mimic mutants. For example, *PR1 (pathogenesis-related 1*) and *PBZ1 (probenazole-inducible 1*) were expressed abundantly in *spl11, spl18* and *HM47* mutants^10^,^11^,^12^,^13^. Hence, studying lesion mimic mutants will help us to elucidate the mechanism associated with leaf senescence and defense responses.

Glutamate synthase (GOGAT) and glutamine synthetase (GS) are known as key enzymes in the process of inorganic nitrogen assimilation^14^,^15^,^16^. Through coordinated action of GOGAT and GS, the inorganic nitrogen is transformed to organic nitrogen^17^. Ammonia, the main form of inorganic nitrogen, is assimilated though GS/GOGAT cycle to produce glutamine (Gln) and glutamate (Glu), both of which constitute the main form of organic nitrogen. In higher plants, there are two kinds of GOGAT^18^, viz. NADH-GOGAT (EC 1.4.7.1) and Fd-GOGAT (EC 1.4.1.14), using NADH and ferredoxin (Fd) as the electron donors respectively. NADH-GOGAT was found mainly in non-photosynthetic tissues such as roots and seeds^19^, while Fd-GOGAT plays a major role in reassimilating the large amounts of ammonia derived from photorespiration^20^. There are two expressed Fd-GOGAT genes in *Arabidopsis*, viz. *AtGLU1* and *AtGLU2*. Analysis of *gls* mutants revealed that the major role of *AtGLU1* is to reassimilate the large amounts of ammonia derived from photorespiration in leaves, while *AtGLU2* functions in the assimilation of primary nitrogen in roots^20^. In another study, authors reported *Arabidopsis* Fd-GOGAT encoded by *GLU1* is dual targeted to the chloroplasts and mitochondria, and plays an important role in photorespiration, largely consistent with the previous study^21^. In rice, Fd-GOGAT was revealed to locate mainly in mesophyll cells of leaves, chloroplast-containing cross-cells of grain pericarp, as well as in the apical meristem through immunocytological analysis^22^. Nevertheless, the function of Fd-GOGAT in leaf senescence and defense response was largely unknown in rice.

Here, we report a new spotted leaf mutant (*spl32*) derived from an *indica* cultivar 9311 by radiation. The *spl32* mutant is covered with necrotic spots in leaves from tip to base in seedling stage, and spotted leaves emerge from bottom to top of the plant. Positional cloning indicated that *SPL32* encodes an Fd-GOGAT protein. We further identified the mutant was subjected to oxidative stress due to suppressed reassimilation system of ammonia, thereby inducing increased resistance to *Xanthomonas oryzae* pv. *Oryzae (Xoo*). Our results reveal that Fd-GOGAT involves leaf senescence and defense response in rice.

## Results

### Phenotypic analyses of the *spl32* mutant

**Figure 1:**
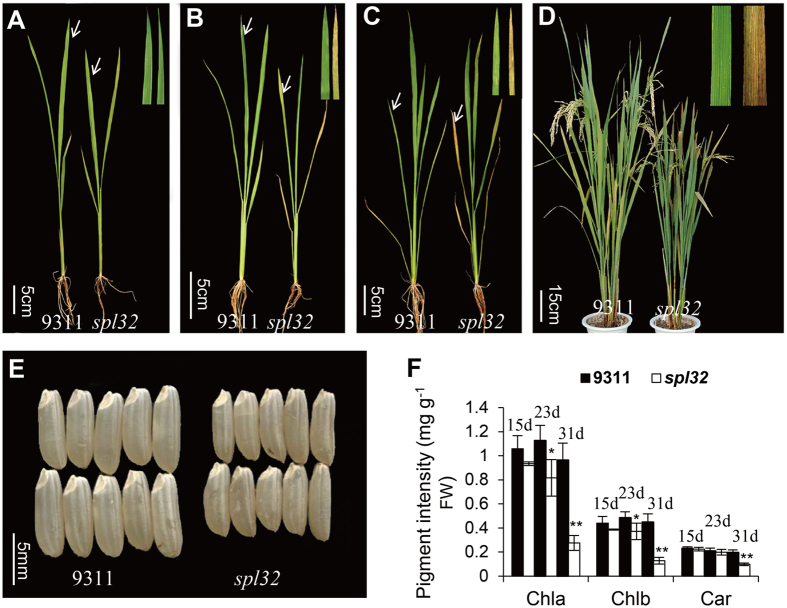
Phenotypic comparison of the wild-type 9311 and spl32 plants. (**A**,**B**,**C**) Phenotypes of 15 (**A**), 23 (**B**), 31 (**C**) days old wild-type 9311 and *spl32* seedlings. The white arrows indicate the fourth real leaves of the wild-type 9311 and *spl32* plants. The inserts show enlargement of the fourth real leaves of the wild-type 9311 and *spl32* mutant (**A**–**C**). (**D**) Phenotypic comparison of the wild-type 9311 and *spl32* mutant at heading stage. The insert shows flag leaves of the wild-type 9311 and *spl32* plants. (**E**) Comparison of the wild-type 9311 and *spl32* seeds (dehulled). Bars = 5 cm in A, B and C, 15 cm in D and 5 mm in (**E**). (**F**) Determination of the chlorophyll contents of the fourth leaves of the wild-type 9311 and *spl32* mutant at three different developmental stages: 15, 23, 31d after sowing. Chla, Chlorophyll a; Chlb, chlorophyll b; Car, total carotenoids. Data represent means ± SD of three independent measurements. Asterisks indicate the statistical significance levels according to Student’s *t* test: **P < 0.01 and *P < 0.05. FW, fresh weight.

Leaves of the *spl32* mutant remained largely the same as the wild type before four-leaf stage (Fig. 1A). The necrotic spots were initiated from tip of older leaves in five-leaf stage (Fig. 1B) and then spread through whole leaf (Fig. 1C). From tillering to heading, the necrotic spots became more serious, accompanied by shorter culm and smaller grain, compared to the wild-type 9311 (Fig. 1D,E). Agronomic traits, such as tiller number, seed setting rate, and thousand kernel weight, were all significantly decreased in the *spl32* mutant (Table 1).

Environmental conditions could induce formation of lesion mimic symptom^23^, thus a shading experiment was conducted here. There was no difference between shaded leaf and non-shaded leaf in the wild type. Remarkably, non-shaded leaf in the *spl32* mutant showed obvious lesion mimic phenotype, in contrast to shaded leaf showing no symptom (Supplemental Fig. S1). Thus the formation of lesion is induced by light in the *spl32* mutant.

According to the severity of lesion spots in the *spl32* mutant, we divided the phenotype into three stages, i.e. before appearance of spots (the first stage), spots emerge (the second stage), and serious spots present (the third stage). Chlorophyll contents were examined in the three stages. The result showed that the contents of chlorophyll *a* and chlorophyll *b* were both decreased during the second and third stages in the *spl32* mutant relative to the wild-type 9311(Fig. 1F).

### Chloroplast changes in leaves of the *spl32* mutant

**Figure 2:**
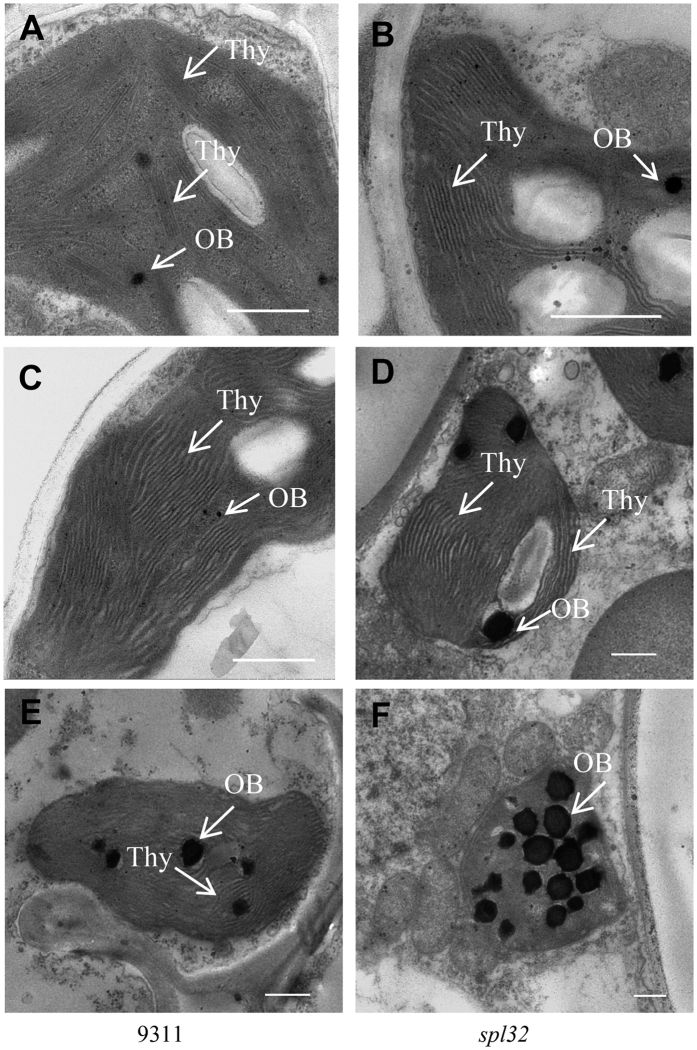
Transmission electron microscopy analyses. The wild-type 9311(**A**,**C**,**E**) and *spl32* (**B**,**D**,**F**) mutant at 15d (**A** and **B**), 23d (**C** and **D**), 31d (**E** and **F**) after sowing. Bars = 5 μm. Thy: thylakoid lamellae; OB: osmiophilic body.

Transmission electron microscopy was used to compare ultrastructure of chloroplasts in the 4^th^ leaves of wild-type and *spl32* seedlings at the all three stages. The mutant leaf had same chloroplast morphology as the wild type at the first stage (Fig. 2A,B), that is, chloroplasts were well-developed with rich lamellae and a small number of osmiophilic bodies. Thus chloroplast development was supposed to be unaffected in the *spl32* mutant. At the second stage, the lamellar structure of chloroplasts began to collapse and osmiophilic bodies were obviously increased in the mutant (Fig. 2C,D). Then the shrinking chloroplasts started to disintegrate and gradually little complete thylakoid structure was observed in the *spl32* mutant. At the third stage, number and size of osmiophilic bodies in the mutant chloroplasts were significantly increased, relative to the wild type (Fig. 2E,F). This result suggested that chloroplasts in the *spl32* mutant are normal during initial development but disintegrated early in later stages.

### Gene expression of leaf senescence in the *spl32* mutant

**Figure 3:**
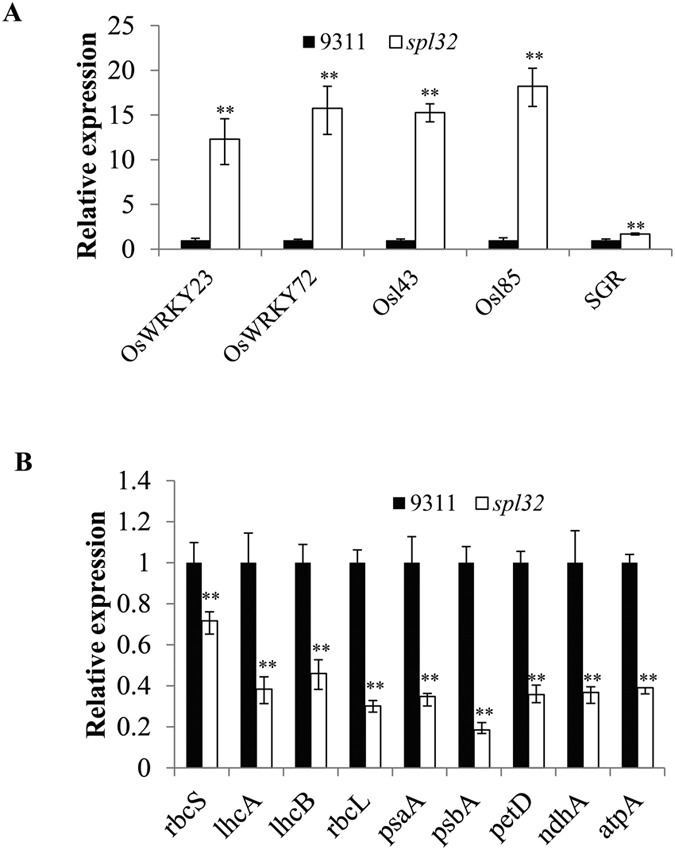
Gene expression analyses. The expression analysis of two senescence-associated transcription factors (*OsWRKY23* and *OsWRKY72*), two senescence-associated genes (*Osl43* and *Osl85) SGR* (**A**) and photosynthesis-associated genes (**B**) at tillering stage. Data represent means ± SD of three independent measurements. Asterisks indicate the statistical significance levels according to Student’s *t* test: **P < 0.01 and *P < 0.05. The accession numbers were listed as follows: *OsWRKY23* BAG98560.1; *OsWRKY23* BAG98549.1; *Osl43* AAK82986; *Osl85* AAL65398; *SGR* AAW82954.1; *rbcS* AAR19268.1; *lhcA* AAB65793.1; *lhcB (Arabidopsis thaliana*), AAA32760; *rbcL* NP_039391.1; *psaA* AJC99402.1; *psbA* AJC99383.1; *petD* AJC99432.1; *ndhA* AJC99458.1; *atpA* AJC99399.1.

Leaf senescence is controlled by a number of genes. Among them, a senescence-induced gene, *STAY GREEN (SGR*), was reported to regulate chlorophyll degradation^24^. Analysis by quantitative RT-PCR (qRT-PCR) revealed that *SGR* transcript was dramatically upregulated in the *spl32* mutant at tillering stage (Fig. 3A). Additionally, many transcription factors and senescence-associated genes (SAGs) were reported upregulated to induce leaf senescence^25^,^26^. To confirm senescence occurred in the *spl32* mutant, we performed gene expression of *OsWRKY23, OsWRKY72, Osl43* and *Osl85* using qRT-PCR (Fig. 3A). The result showed that expression levels of all four genes were notably increased in the mutant at tillering stage (Fig. 3A), in agreement with the symptom of early leaf senescence.

A previous study showed photosynthesis-associated gene expressions were decreased when leaf senescence appeared^5^. Thus nine photosynthesis-associated genes were selected for analysis by qRT-PCR (Fig. 3B). Among them, three (*rbcS, lhcA,* and *lhcB*) belong to nuclear-encoded genes and the others (*rbcL, psaA, psbA, petD, ndhA,* and *atpA*) are chloroplast-encoded ones. The result revealed that all photosynthesis-related genes were down-regulated in *spl32* mutant leaves (Fig. 3B).

### Map-based Cloning of the *SPL32* gene

**Figure 4:**
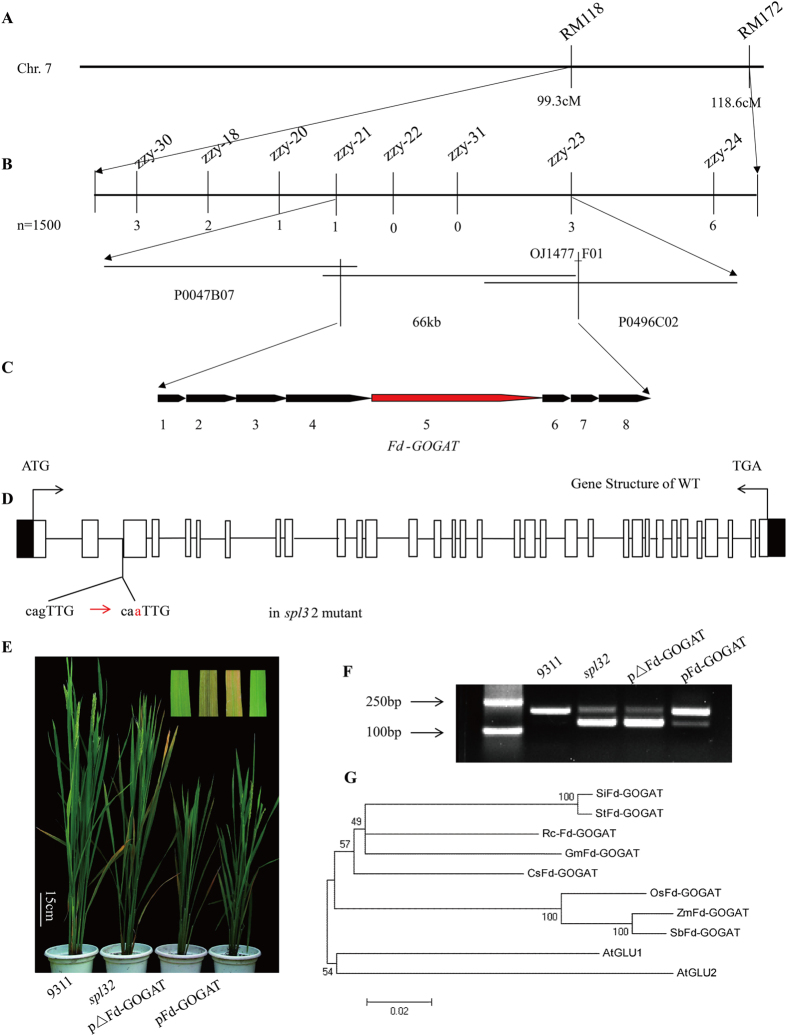
Cloning of *SPL32* gene and phylogenetic analysis of Fd-GOGAT homologs. (**A**) The *SPL32* locus was mapped to the long arm of chromosome 7 between the markers RM118 and RM172. (**B**) Mapping of the *SPL32* locus between markers zzy-21 and zzy-23 based on bacteriophage P1-derived artificial chromosome (PAC) or bacterial artificial chromosome (BAC) clone sequence (PAC1, P0047B07; BAC2, OJ1477_F01; PAC3, P0496C02). ‘n’ represents homozygous recessive individuals derived from an F_2_ population and an F_2:3_ population from the cross *spl32* × 02428. The number of recombinants is indicated below the map. (**C**) the *SPL32* locus was narrowed down to a 66-kb region; there are 8 putative open reading frames (ORFs). (**D**) The gene structure of 9311. The junction of the third exon and intron had a single base mutation (G into A) in ORF4 of *spl32* mutant. (**E**) Complementation analysis of the *spl32* mutant. The wild-type 9311 plant and homozygous recessive mutant derived from the cross *spl32* × Nipponbare transformed with pFd-GOGAT show normal green leaves, whereas the *spl32* mutant and homozygous recessive mutant of F2 population transformed with pΔFd-GOGAT show necrotic spots in leaves. (**F**) PCR analysis with primers Zf-1 showed the difference in the *Fd-GOGAT* cDNA in the wild-type 9311, *spl32* mutant, ΔFd-GOGAT transgenic plants and Fd-GOGAT transgenic plants. Alternative splicing occurs in the coding region of *Fd-GOGAT* in *spl32* mutant. (**G**) Phylogenetic analysis of Fd-GOGAT. Fd-GOGAT is most closely homologous to maize and sorghum Fd-GOGATs. The accession numbers were listed as follows: SiFd-GOGAT (*Solanum lycopersicum*), XP_004234830.1; StFd-GOGAT (*Solanum tuberosum*), XP_006363768.1; RcFd-GOGAT (*Ricinus communis*), XP_002526914.1; GmFd-GOGAT (*Giycine max*), XP_006576787.1; CsFd-GOGAT (*Cucumis sativus*), XP_004136778.1; OsFd-GOGAT (*Oryza sativa*), NP_001060520.1; ZmFd-GOGAT (*Zea mays*), NP_001105693.1; SbFd-GOGAT (*Sorghumbicolor*), XP_002463318.1; AtGLU1 (*Arabidopsis thaliana*), NP_850763.1; AtGLU2 (*Arabidopsis thaliana*), NP_181655.1.

Genetic analysis from reciprocal crosses between 9311 and the *spl32* mutant showed that the lesion mimic phenotype was controlled by a single recessive nuclear locus (Supplemental Table S1). Molecular analysis of an F_2_ population and its derived F_2:3_ population from the cross *spl32* × 02428 (a *japonica* rice variety) restricted the *SPL32* locus between the markers RM118 and RM172 on chromosome 7 (Fig. 4A). Twelve insertion/deletion (InDel) markers were developed between the two markers (Supplemental Table S2). The *SPL32* locus was further narrowed down to a 66-kb region (Fig. 4B), containing eight putative open reading frames (ORFs) (Fig. 4C). Sequencing all ORFs indicated a single base mutation (G into A) in the junction between the third exon and intron in *Fd-GOGAT (Os07g0658400*) (Fig. 4D) led to alternative splicing in mRNA. Thus a 57-bp sequence was missing in the complementary DNA (cDNA) in the mutant (Fig. 4F). A newly developed InDel marker zf-1 was used to distinguish the wild type from *spl32* mutant genotypes by the size of amplified cDNA fragment (Supplemental Table S3). The cDNA of *spl32* mutant has two forms: one is supposed to encode a truncated protein (ΔFd-GOGAT) (a strong band) lacking 19 amino acids; and the other one encodes a whole Fd-GOGAT protein (indicated by a weak band), just like wild type (Fig. 4F). Therefore the *spl32* mutant is an *Fd-GOGAT* knockdown mutant.

To verify identity of Fd-GOGAT, we constructed the plasmid pFd-GOGAT, containing a 5-kb fragment with only *Fd-GOGAT* coding region of 9311. We also constructed the plasmid pΔFd-GOGAT, containing the 57 bp-deleted fd-gogat coding region of the mutant. Then, we introduced the two constructs into homozygous recessive individuals selected from the cross *spl32* × Nipponbare. All transgenic lines containing pFd-GOGAT rescued the lesion mimic phenotype, whereas 10 independent lines transformed with vector pΔFd-GOGAT didn’t (Fig. 4E,F). The result indicated that the 57-bp missing in *Fd-GOGAT* cDNA was responsible for the necrotic spots in the *spl32* mutant. Phylogenetic analysis showed that rice Fd-GOGAT was most closely related to maize and sorghum Fd-GOGATs (Fig. 4G).

### Expression analyses and subcellular localization of Fd-GOGAT protein

**Figure 5:**
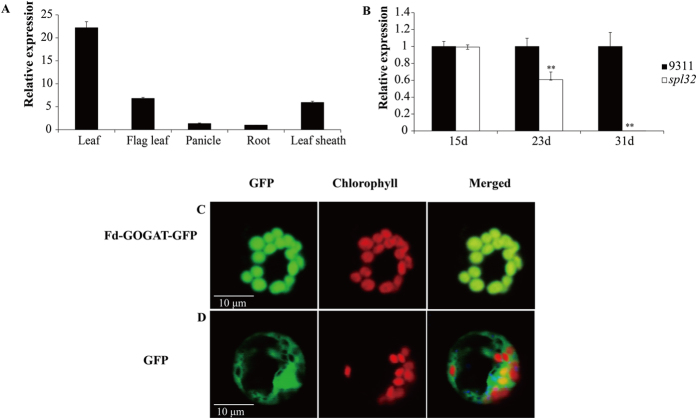
Expression analysis and subcellular localization of Fd-GOGAT protein. (**A**) The expression of *Fd-GOGAT* in different tissues. Leaf was selected from seedling of 30 days after sowing of the wild-type 9311; root, flag leaf, panicle, and leaf sheath were selected from the wild-type 9311 at heading stage. (**B**) The expression of *Fd-GOGAT* at three different developmental stages. The fourth leaves of 15, 23, 31 days after sowing in wild-type 9311 and *spl32* mutant were selected. Data are means ± SD (n = 5). Asterisks indicate the statistical significance levels according to Student’s *t* test: **P < 0.01 and *P < 0.05. (**C**) GFP signals of the Fd-GOGAT-GFP fusion protein located in the chloroplasts of rice protoplasts. (**D**) GFP signals were dispersed in rice protoplasts. Bars = 10 μm.

To investigate expression pattern of *Fd-GOGAT* in rice, we analyzed *Fd-GOGA*T expression in different tissues by qRT-PCR. We found that *Fd-GOGAT* was constitutively expressed in leaf, root, panicle and other tissues analyzed. The *Fd-GOGAT* was most pronounced in young leaves at seedling stage while relatively weak in roots (Fig. 5A), consistent with a previous study that *Fd-GOGAT* mainly expressed in photosynthetic tissues^16^. To detect expression levels of *Fd-GOGAT* at the three different developmental stages in the seedling period, we chose the fourth leaves of the *spl32* mutant and 9311 at 15, 23, and 31 days after sowing (DAS) for qRT-PCR analysis. As the development of lesion spots, *Fd-GOGAT* expression showed a decreasing trend. At 31 DAS when the fourth leaves of *spl32* mutant were filled with necrotic spots, *Fd-GOGAT* almost couldn’t be detected in the mutant (Figs 1C and 5B), indicating that the more severe necrotic spots, the lower expression of Fd-GOGAT in the *spl32* mutant.

To further investigate subcellular localization of Fd-GOGAT protein in rice, we use ChloroP^27^ and TargetP^28^ to search subcellular localization signals. We found that N-terminus of Fd-GOGAT protein comprised a putative chloroplast-targeting signal. Thus, we constructed a fusion protein with Fd-GOGAT and green fluorescent protein (GFP), and transformed it into rice protoplasts. Confocal microscopy was used to observe the fluorescent signals in 16–18 h after transformation. We observed that green fluorescent signal from Fd-GOGAT-GFP was colocalized with the autofluorescent signals of chlorophylls in the chloroplasts (Fig. 5C,D), suggesting Fd-GOGAT protein is located in the chloroplasts.

### The reassimilating of ammonia is inhibited in the *spl32* mutant

**Figure 6:**
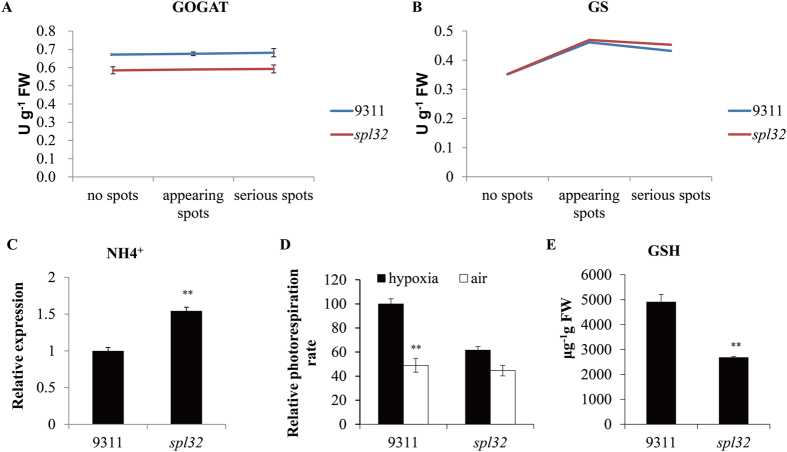
Analysis of GOGAT and GS activities, determination of photorespiration rate, contents of ammonia (NH_4_^+^) and glutathione (GSH). (**A** and **B**) Enzyme activity of GOGAT and GS in wild-type 9311 and *spl32* mutant, non-spotted, spot-appearing and serious-spotted leaves of *spl32* mutant and the corresponding sections of leaves in the wild-type 9311 at tillering stage. (**C**) Determination of NH_4_^+^ content in the wild-type 9311 and *spl32* mutant at tillering stage. (**D**) Determination of net photosynthesis rate in atmospheric condition and hypoxia conditions using portable photosynthesis system LI-6400XTOPEN6.1. At tillering stage, new-fully expanded leaves of wild-type 9311 and *spl32* mutant were chosen and net photosynthesis rate of mid-blade was measured before 12 o’clock. (**E**) Determination of GSH content in the wild-type 9311 and *spl32* mutant at tillering stage. Data represent means ± SD of three independent measurements. Asterisks indicate the statistical significance levels according to Student’s *t* test: **P < 0.01 and *P < 0.05. FW, fresh weight.

In photosynthetic green leaves and shoots of *Arabidopsis*, Fd-GOGAT accounts for more than 96% of total enzyme activity and the remaining is derived from NADH-GOGAT^29^. Here we measured GOGAT activity in 9311 and the *spl32* mutant (Fig. 6A). The result revealed that GOGAT activities in mutant leaves were decreased by 13.2% in non-spotted stage, 12.7% in spot-appearing stage and 13.2% in serious-spotted stage, respectively. We also evaluated effect of the decreased GOGAT activity on GS expression by ELISA Kit (Fig. 6B). It showed that GS activity in the mutant had no difference from 9311 in the all three stages.

Besides, GOGAT and GS were known as key enzymes involved in inorganic nitrogen assimilation^14^,^15^,^16^. To investigate amino acid changes in the *spl32* mutant, we measured free amino acid contents in 9311 and the *spl32* mutant at tillering stage (Table 2). Many amino acids such as Glu, Ser, Asp, Gly, Ala, Val, Met, Ile, Leu, Tyr, Phe, Lys, and His were significantly decreased, while Gln, Cys and Arg were dramatically increased in the *spl32* mutant. Since Glu and Gln are produced from ammonia though GS/GOGAT cycle and both amino acids are significantly altered in the *spl32* mutant, so we determined ammonia content at the tillering stage and found ammonium was noticeably increased in the mutant (Fig. 6C).

Previous study indicated Fd-GOGAT plays a major role in photorespiration by reassimilating ammonia^20^. To evaluate effect of the increased ammonia content on photorespiration, we measured photorespiration rates by supplying hypixic gas (<2%) to wild-type and mutant leaves, as described previously^30^. The result showed the *spl32* mutant had much lower net photosynthetic rate than its wild type under hypoxic conditions, but no difference under atmospheric conditions (Fig. 6D).

Generally photorespiration can affect synthesis of glutathione (GSH), which is produced from condensation reaction of Gly and γ-glutamylcysteine^31^. Consistent with decreased Gly (Table 2), GSH content was reduced in the *spl32* mutant (Fig. 6E).

### Antioxidant enzyme activities and active oxygen burst in the *spl32* mutant

**Figure 7:**
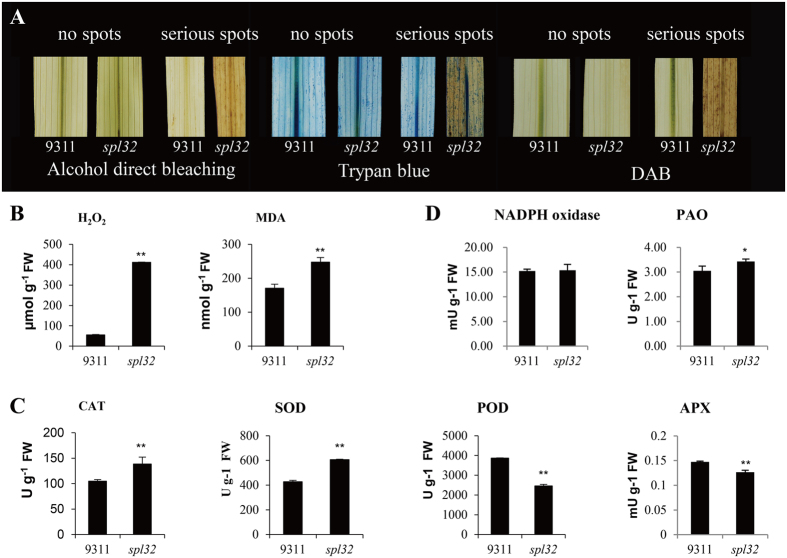
Dying experiments and the determination of enzyme activities involved in scavenging and generating mechanism of ROS. (**A**) non-spotted leaves and serious spotted leaves of *spl32* mutant and the corresponding sections of leaves in the wild-type 9311 at tillering stage were stained by trypan blue and 3, 3-diaminobenzidine (DAB) respectively. Controls were not stained and decolorized directly by alcohol. (**B**) The contents of hydrogen peroxide (H_2_O_2_) and malonaldehyde (MDA) in the wild-type 9311 and *spl32* mutant at tillering stage. FW, fresh weight. (**C**) The enzyme activities of catalase (CAT), superoxide dismutase (SOD), ascorbate peroxidase (POD), and peroxidase (APX) in wild-type 9311 and *spl32* mutant at tillering stage. FW, fresh weight. (**D**) The enzyme activities of NADPH oxidase and polyamine oxidase (PAO) in wild-type 9311 and *spl32* mutant at tillering stage. FW, fresh weight. Data represent means ± SD of three independent measurements. Asterisks indicate the statistical significance levels according to Student’s *t* test: **P < 0.01 and *P < 0.05. FW, fresh weight.

GSH is an important active substance in plants and participates in formation of disulphide, sulfur ether and thioester. Meanwhile, it can remove free radicals to scavenge their poison in plants^32^. Hence we determined reactive oxygen species (ROS) content in plant cells by trypan blue dyeing. Leaves with no spots and serious spots in *spl32* mutant and the corresponding parts of leaves in the wild type were stained at tillering stage (Fig. 7A). The result showed that no dyeing was observed on non-spotted leaves from either the mutant or wild type. In contrast, leaves with serious spots in *spl32* mutant were dyed blue. This result indicated that severe cell necrosis occurred in *spl32* mutant when spots emerged.

To determine accumulation level of hydrogen peroxide in the *spl32* mutant, similar leaves in the above treatment were stained with 3,3-diaminobenzidine (DAB). No staining was detected in the non-spotted leaves in both the *spl32* mutant and 9311; nevertheless, leaves with serious spots in the *spl32* mutant were dyed bronzing (Fig. 7A). Moreover, we determined hydrogen peroxide contents at tillering stage, and found that *spl32* mutant leaves accumulated much more hydrogen peroxide than the wild type (Fig. 7B).

Malonaldehyde (MDA) is one of the most important products of membrane lipid peroxidation, and its accumulation can aggravate membrane damage, indirectly reflecting the degree of cellular damage^33^. Hence, we measured MDA content at tillering stage (Fig. 7B). As expected, the *spl32* mutant showed significantly increased MDA in leaves, compared to the wild type. Thus the *spl32* mutant tissue could be susceptible to oxidative damage and started senescence more quickly than the wild type.

A previous study showed that enzymes involved in antioxidant systems were expressed in lesion mimic mutants^34^. Here, we detected activities of four oxidative stress related enzymes, including catalase (CAT), superoxide dismutase (SOD), ascorbate peroxidase (APX), and peroxidase (POD), respectively. As shown in Fig. 7C, activities of CAT and SOD were significantly increased while POD and APX activities were remarkably decreased in the mutant. Meanwhile, two other enzymes that catalyze ROS generation, viz. NADPH oxidase and polyamine oxidase (PAO), were also determined (Fig. 7D). It revealed that activity of PAO was increased while NADPH oxidase showed no change in the *spl32* plants.

### The *spl32* mutant has enhanced disease resistance and up-regulated PR marker genes

**Figure 8:**
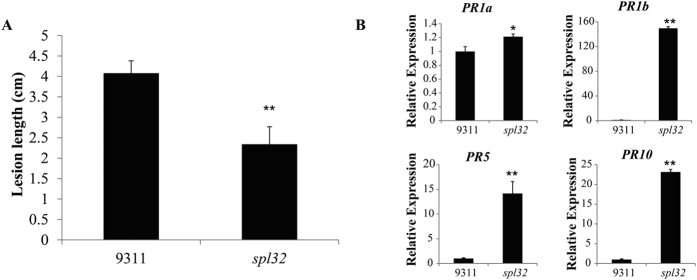
Detection of bacterial blight pathogen resistance and expression of resistance related genes. (**A**) Lesion lengths were determined after plant leaves inoculated by bacterial blight pathogen zhe173. Bars represent ± SD of six replicates. (**B**) The expression of pathogenesis-related (*PR*) marker genes at tillering stage. Bars represent ± SD of three measurements. The gene numbers of *PR* genes are as follows: *PR1a*, AJ278436; *PR1b*, B109D03; *PR5*, X68197; *PR10*, D38170. Asterisks indicate the statistical significance levels according to Student’s *t* test: **P < 0.01 and *P < 0.05.

The appearance of necrotic spots in the *spl32* mutant resembles hypersensitive response (HR), which constitutes an important resistance mechanism in plants^35^. To evaluate response of the *spl32* mutant to pathogen, bacterial blight pathogen *Xoo* strain Zhe173 was used for inoculation. As shown in Fig. 8A, the *spl32* plants demonstrated significantly enhanced resistance compared to its wild type.

Previous studies indicated that oxygen stress not only activated protection mechanism but also acted as a signal factor in undamaged tissues to enable systemic acquired resistance (SAR)^36^,^37^. So we detected expressions of four pathogenesis-related (*PR*) marker genes (*PR1a, PR1b, PR5* and *PR10*) associated with defense response. The results revealed that all *PR* genes were consistently up-regulated in the *spl32* mutant (Fig. 8B).

## Discussion

Early leaf senescence occurred in *spl32* mutant, confirmed by decreased pigment content and increased MDA content and SOD activity as physiological indicators, chloroplast degradation as cellular indicators, and both upregulation of senescence transcription factors (*OsWRKY23* and *OsWRKY72*) and senescence-associated genes (*Osl43* and *Osl85*) and downregulation of photosynthesis-related genes as molecular evidence. ROS plays an important role in early leaf senescence^38^. The main sources of ROS are from NADPH oxidase and PAO^36^. In the *spl32* mutant, activity of NADPH oxidase showed no change but PAO activity was increased compared to the wild type. NADPH oxidase converts molecular oxygen into superoxide anion, and PAO interconverts polyamines, producing hydrogen peroxide. Overexpression of *AtPAO3* resulted in an increased production of both hydrogen peroxide and superoxide anion^39^. Here, a large amount of hydrogen peroxide were accumulated, which thereafter induced CAT activity; while superoxide anion did not accumulate in the *spl32* mutant by nitrotetrazolium blue chloride test (Supplemental Fig. S2), which might be that the increase of superoxide anion was promptly eliminated by SOD. Moreover, photorespiration provides Gly for GSH synthesis^31^ and plays a major role in the readjustment of redox homeostasis^40^. We found photorespiration was dramatically inhibited by over-accumulation of ammonium in the *spl32* mutant. Meanwhile, contents of Gly and GSH were both decreased, accompanied by extremely decreased activities of POD and APX in the *spl32* mutant. Therefore, the ROS accumulated in the mutant can probably be attributed to overproduction of hydrogen peroxide and damaged scavenging pathway.

*OsWRKY23* played an important role in senescence and resistance, and overexpression of *OsWRKY23* enhanced expression of PR related genes and increased resistance to the bacterial pathogen *Pseudomanas syringae* in *Arabidopsis*^41^. In the *spl32* mutant, disease resistance to *Xoo* was significantly enhanced, consistent with a recently published study showing Fd-GOGAT plays an important role in broad spectrum bacterial blight resistance^42^. Moreover, we found four *PR* marker genes were up-regulated in the *spl32* mutant. There are two major signaling pathways in plant disease resistance, viz. salicylic acid (SA) and jasmonic acid (JA)^43^. Since expressions of *PR1a* and *PR1b* were both enhanced in the mutant, suggesting the *SPL32* might involve in SA and JA signal transduction.

Through coordinated action of GS and GOGAT, inorganic nitrogen is transformed to organic nitrogen^20^. GS catalyzes ammonium into Glu, thereby producing Gln. Subsequently, GOGAT transfers amide amino group of Gln to 2-oxoglutrate, forming two molecules of Glu again^22^. In this study, as contents of ammonium and Gln were remarkably increased, while Glu was decreased in the *spl32* mutant, we concluded that Glu could be effectively catalyzed into Gln by functionally normal GS in the mutant. In contrast, decrease of GOGAT activity due to the *spl32* mutation caused the over-accumulation of Gln. In addition to changed contents of Glu and Gln, we also found many other free amino acids, except Cys and Arg, were decreased in *spl32* mutant, thus it was intriguing to investigate whether and how Fd-GOGAT involves in a complex cross-pathway regulatory mechanism in amino acid metabolism.

It is noteworthy that photorespiration plays an important role in protecting photosynthetic organs from damage through excessive absorption of light energy^44^. In the *spl32* mutant, the lesion was induced by light and photorespiration rate was reduced, therefore light might be a trigger to accelerate oxidative damage in leaves, compared to the wild type.

In *Arabidopsis*, Fd-GOGAT contributes to major GOGAT enzyme activity, and the remaining is derived from NADH-GOGAT^20^. There are two *NADH-GOGAT* genes in rice^19^. *OsNADH-GOGAT1* is mainly expressed in growing tissues, such as immature leaves, the early development of spikelet and roots, while *OsNADH-GOGAT2* mRNA is only expressed in leaves and leaf sheathes. These isoforms have distinct functions: OsNADH-GOGAT1 is essential in assimilation of ammonia in roots^45^; OsNADH-GOGAT2 plays an important role in remobilization of nitrogen during senescence of rice leaves^46^. In this study, we found transcript levels of the *Fd-GOGAT* gene were dramatically reduced at 23 DAS and nearly abolished at 31 DAS in the *spl32* mutant plants (Fig. 5C), whereas the GOGAT enzyme activity was only reduced constantly by 13% (Fig. 6A) during the period. The discrepancy between transcript levels and enzyme activities is possible due to following reasons: (1) potential post-transcriptional and post-translational regulation of *Fd-GOGAT*, such as alternative splicing, RNA stability, protein stability and modification, etc.; (2) compensation of another rice Fd-GOGAT, viz. osNADH-GOGAT. In the *spl32* mutant, we indeed observed when expression level of *Fd-GOGAT* fell down to the lowest level at the most serious period of spots, *NADH-GOGAT2* had a dramatic increase at the same stage (Supplemental Fig. S3). Therefore we deduce NADH-GOGAT2 might partially but not completely, compensate the function of Fd-GOGAT.

In conclusion, we report herein that *SPL32* encodes an Fd-GOGAT that is highly expressed in young leaves. In the *spl32* mutant, reassimilation of Gln and ammonia is inhibited due to *Fd-GOGAT* knockdown. Photorespiration rate and Gly content are decreased as well as glutathione content and some antioxidant enzyme activities, resulting in uncontrolled oxidative stress in the mutant. Moreover, defense response genes are activated and disease resistance is enhanced in the *spl32* mutant. This provides a prospect in developing new rice resistant lines by manipulating *SPL32* in gene engineering.

## Materials and Methods

### Plant materials and growth conditions

The rice lesion mimic mutant *spl32* was selected from an M2 population of 9311 induced by ^60^Co radiation. M2 seeds were grown in a paddy field under natural growing conditions in Rice Station, Nanjing Agricultural University, Nanjing, China. The mutant was stabilized through backcrossing with 9311 and selection for three times.

### *SPL32* gene cloning

For genetic analysis, F_2_ populations were generated from two crosses of 9311/*spl32* and *spl32*/9311. In both F_2_ populations, segregation of green-leaf and spotted-leaf individuals fitted a 3:1 ratio. To map the *SPL32* gene, we constructed an F_2_ mapping population generated from a cross *spl32* × 02428 (a *japonica* rice variety). Totally 330 SSR markers evenly spanning 12 rice chromosomes were used to rough map *SPL32.* The *SPL32* was initially mapped to the markers between RM118 and RM172 on chromosome seven using 588 F_2_ mutant plants. Afterwards, the *SPL32* locus was further narrowed down between markers zzy-21 and zzy-23 using 801 F_2:3_ mutant plants. The mapping primers were listed in Supplemental Table S2. The target region was analyzed with RiceGAAS (http://ricegaas.dna.affrc.go.jp) and eight ORFs were predicted. Then coding sequences of eight ORFs in both 9311 and *spl32* mutant were amplified for sequencing.

### Complementation of the *spl32* mutant

To create complementation construct pFd-GOGAT, cDNA of *Fd-GOGAT* was amplified by RT-PCR from 9311 with the primer pairs listed in Supplemental Table S3. The resulting fragments were inserted into the vector 1300–221-FLAG in which the *Fd-GOGAT* gene was under the control of 35S promoter. Since transformation events with 9311 as receptor was difficult, then the construct was introduced into some F_2_ mutant plants selected from a cross between Nipponbare and *spl32* mutant, by *Agrobacteriun tumefaciens*-mediated method as described previously^47^.

### Determine of pigment contents

Pigment contents were measured according to the method described previously^48^. Fresh leaves of *spl32* mutant plants and 9311 at three different developmental stages (15, 23, and 31 DAS) were collected. Absorption values were measured using UV/Vis spectrophotometer (Beckman DU800 USA).

### Transmission electron microscopy

Samples of 9311 and *spl32* mutant leaves were prepared for electron microscopy from the fourth leaves of seedling after growing 15, 23, 31 days under standard field conditions. The collected leaves were cut into small pieces and fixed in 2.5% glutaraldehyde in a phosphate buffer at 4 °C for 4 h, rinsed and incubated overnight in 1% OsO4 at 4 °C, dehydrated by an ethanol series, and infiltrated with a series of epoxy resin, and then embedded in Spurr’s medium prior to thin sectioning. Sections were stained again using uranyl acetate and measured with a JEM-1230 electron microscope.

### RT-PCR and Real-Time RT-PCR

Total RNA of rice leaves was extracted with a RNA Prep Pure Plant kit (Tiangen) following the manufacturer’s instructions. Each RNA sample (2 μg) was reverse transcribed using the QuantiTect reverse transcription kit (Qiagen). Primer pairs were designed using Primer Express (Applied Biosystems) and listed in Supplemental Table S3. Additionally, the primer information of some genes including *STAY GREEN (SGR*), two senescence-associated transcription factors, senescence-associated genes and photosynthesis-associated genes were referred from a previous study^49^. Real-time PCR analysis was performed using ABI7300HT fast real-time PCR system with the SYBR Premix Ex Taq (TaKaRa; catalog no. RR041A). For each sample, we performed real-time PCR with three technical replicates on three biological replicates. *ACTIN* gene in rice was used as an internal control.

### Amino acid sequence alignment and phylogenetic relationship

Searching and downloading the homologous proteins of Fd-GOGAT in different species were carried out in the NCBI website, and then homologous proteins of Fd-GOGAT were analyzed using biological software BioEdit7.0. Multiple sequence alignment was conducted using ClustalW program^50^, and phylogenetic analysis method described previously^51^.

### Subcellular location of GFP protein

To investigate subcellular location of Fd-GOGAT, coding sequences of *Fd-GOGAT* were amplified by PCR using primer pairs listed in Supplemental Table S3. PCR products were cloned into pAN580 vector. The vector was transformed into rice protoplasts and incubated in the dark at 24 °C for 14–16 h according to the method described previously^52^. Finally, GFP fluorescence was observed using a confocal laser scanning microscopy (Zeiss LSM780).

### Shading experiment and photorespiration determination

At heading stage, flag leaves (leaves were all green and no spots) in the *spl32* mutant and 9311 were wrapped in tinfoil, then the plants were grown in the paddy filed for 10 days under natural light. Five flag leaves (each from mutant and wild type) were randomly selected for treatment. To investigate photorespiration in the *spl32* mutant, we measured net photosynthesis rate in atmospheric and hypoxia condition using portable photosynthesis system LI-6400XTOPEN6.1. At tillering stage, we chose new-fully expanded leaves of the *spl32* mutant and its wild type, and measured net photosynthesis rate of mid-blade before 12 o’clock. In both mutant and wild type, 10 plants were randomly selected. The gas access of instrument for measuring photosynthesis was input with 98% nitrogen and 2% oxygen to produce hypoxia conditions. Net photosynthesis rates were examined according to the manufacturer’s recommendation.

### Determination of various antioxidant indexes

Commercial kits for assaying contents of GSH, hydrogen peroxide, and MDA, as well as enzyme activities for CAT, SOD, and POD were all purchased from Biological Engineering Institute of Nanjing Jiancheng^TM^. The enzyme activities of APX, PAO and NADPH oxidase were determined by corresponding Enzyme-Linked Immunosorbnent Assay (ELISA) Kit purchased from Biological Engineering Institute of Jiangsu lyuye^TM^. Determination experiments were conducted at tillering stage. All determination protocols referred to manufacture’s instruction. In presence of peroxidases, DAB and hydrogen peroxide could react quickly and form brown polymer deposition. Integrity of cell membrane was examined by trypan blue. Dyeing experiments with trypan blue and DAB were performed according to Wang *et al*.^53^ using leaves with non-spotted and serious spotted leaves of *spl32* mutant respectively, and their corresponding sections of normal leaves from 9311 at tillering stage. Finally, the leaves were photographed after the decolorization.

### Assays of GOGAT and GS activities

GOGAT were determined by GOGAT ELISA Kit purchased from the company of Shanghai Yansheng^TM^ and GS activities by GS ELISA Kit from Biological Engineering Institute of Jiangsu lyuye^TM^. We selected leaves from different sections of 9311 and *spl32* plants (carrying no spots, newly-appearing spots, and serious spots) at tillering stage. Each sample was repeated three times according to the manufacturer’s instructions.

### Inoculation with bacterial blight pathogen

*Xoo* strain Zhe173 (race IV) was provided by Jiangsu Academy of Agricultural Science. New-fully expanded leaves of six independent *spl32* plants and 9311 at the tillering stage were inoculated with Zhe173 suspensions (optical density of 0.5 at 600 nm) using clipping leaf method^54^. Lesion lengths on inoculated plants were measured 21 days after inoculation.

### Free amino acids and ammonium content analyses using automatic amino acid analyzer

Free amino acids and ammonium extraction method is as follows: At tillering stage, we chose new-fully expanded leaves of the *spl32* mutant and 9311. Samples corresponding to ~300 mg fresh weight (FW) were collected and ground to powders in liquid nitrogen. We also set a blank control without samples. Aliquots of 100 mg FW and blank control were added into 10 mL of 0.1 mol/L HCl, then sealed the lid and properly soaked with shaking. The mixture was extracted by an ultrasonic equipment at temperature 40–50 °C. After 1–1.5 h, the samples were cooled and centrifuged (10 min at 8000 rpm). The proper amount of supernatant was added into equal volume of 10% sulfosalicylic acid. After cooling for an hour at 4 °C, the samples were centrifuged for 30 min at 15,000 rpm, then pH of the supernatant was adjusted with 8 mol/L NaOH to 2.0. Finally, the samples were filtered by 0.45 μm membrane filters and then determined by High Speed Amino Acid Analyzer (Hitachi L-8800). For ammonium content determination, absorbance was measured at 570 nm when retention time was 72.68 min.

## Additional Information

**How to cite this article:** Sun, L. *et al*. Isolation and characterization of a *spotted leaf 32* mutant with early leaf senescence and enhanced defense response in rice. *Sci. Rep.* **7**, 41846; doi: 10.1038/srep41846 (2017).

**Publisher's note:** Springer Nature remains neutral with regard to jurisdictional claims in published maps and institutional affiliations.

## Supplementary Material

Supplementary Figures and Tables

## Figures and Tables

**Table 1:** Comparison of agronomic traits between wild-type 9311 and *spl32* mutant.

Agronomic traits	9311	*spl32*
Plant height (cm)	119.7 ± 3.6	105.3 ± 3.5^**^
Tiller number per plant	7.4 ± 0.6	5.9 ± 0.6^**^
Flag leaf length (cm)	29.28 ± 2.54	28.02 ± 2.66
Spike length (cm)	22.36 ± 1.66	22.14 ± 1.65
Seed setting rate (%)	92.68 ± 2.30	82.77 ± 6.89^**^
Thousand kernel weight (g)	30.33 ± 0.23	26.33 ± 0.18^**^

Data are presented as means ± SD. Three independent plants were measured to calculate the means value. Statistically significant differences from the respective wild type at **P < 0.01 were detected using *t*-tests.

**Table 2:** Free amino acid content in leaves of wild-type 9311 and *spl32* mutant.

Amino acids (μg/g)	9311	*spl32*
Asp	243.76 ± 11.82	160.32 ± 7.97**
Asn	31.01 ± 0.67	28.71 ± 0.67*
Thr	62.62 ± 7.59	64.48 ± 3.23
Ser	284.68 ± 24.49	101.42 ± 8.84**
Glu	1165.32 ± 60.84	890.08 ± 19.41**
Gln	28.02 ± 0.26	32.18 ± 0.90**
Gly	251.72 ± 3.83	56.68 ± 5.78**
Ala	223.60 ± 10.41	91.74 ± 8.72**
Cys	7.64 ± 1.40	22.78 ± 0.50**
Val	126.94 ± 6.37	55.54 ± 2.64**
Met	201.24 ± 18.65	159.96 ± 3.04*
Ile	46.30 ± 0.98	14.48 ± 1.49**
Leu	73.90 ± 1.63	24.78 ± 2.22**
Tyr	133.42 ± 5.42	63.96 ± 2.70**
Phe	82.42 ± 2.22	34.02 ± 0.52**
Lys	63.02 ± 7.96	31.78 ± 1.42**
His	40.84 ± 1.72	27.04 ± 2.22**
Arg	16.18 ± 0.67	17.88 ± 0.48*
Pro	18.73 ± 0.61	17.59 ± 1.18

Data are presented as means ± SD. Three independent plants were measured to calculate the means value. Statistically significant differences from the respective wild type at *P < 0.05 and **P < 0.01 were detected using *t*-tests.

## References

[b1] Lutts S., Kinet J. M. & Bouharmont J. NaCl-induced senescence in leaves of rice (*Oryza sativa L*.) cultivars differing in salinity resistance. Ann. Bot. 78, 389–398 (1996).

[b2] Pourtau N. . Interactions of abscisic acid and sugar signaling in the regulation of leaf senescence. Planta. 219, 765–772 (2004). 15118859 10.1007/s00425-004-1279-5

[b3] Munns R. Genes and salt tolerance: bringing them together. New Phytol. 167, 645–663 (2005). 16101905 10.1111/j.1469-8137.2005.01487.x

[b4] Céline M. D. . Genetic variation suggests interaction between cold acclimation and metabolic regulation of leaf senescence. Plant Physiol. 143, 434–446 (2007). 17098848 10.1104/pp.106.091355 PMC1761960

[b5] Lim P. O., Kim H. J. & Nam H. G. Leaf senescence. Ann. Rev. Plant Biol. 58, 115–136 (2007). 17177638 10.1146/annurev.arplant.57.032905.105316

[b6] Jiao B. B. . A novel protein RLS1 with NB-ARM domains is involved in chloroplast degradation during leaf senescence in rice. Mol Plant. 5, 205–217 (2012). 21980143 10.1093/mp/ssr081

[b7] Qiao Y. L. . *SPL28* encodes a clathrin-associated adaptor protein complex 1, medium subunit micro 1 (AP1M1) and is responsible for spotted leaf and early senescence in rice (*Oryza sativa*). New Phytol. 185, 258–274 (2010). 19825016 10.1111/j.1469-8137.2009.03047.x

[b8] Wu C. J. . Rice lesion mimic mutants with enhanced resistance to diseases. Mol Genet Genomics. 279, 605–619 (2008). 18357468 10.1007/s00438-008-0337-2

[b9] Mizobuchi R. . Differential expression of disease resistance in rice lesion-mimic mutants. Plant Cell Rep. 21, 390–396 (2002).

[b10] Feng B. H. . Characterization and genetic analysis of a novel rice spotted-leaf mutant *HM47* with broad-spectrum resistance to *Xanthomonas oryzae* pv. *oryzae*. J Integr Plant Biol. 55, 473–483 (2013). 23210861 10.1111/jipb.12021

[b11] Kim S. T. . Proteomics analysis of rice lesion mimic mutant (*spl1*) reveals tightly localized probenazole-induced protein (PBZ1) in cells undergoing programmed cell death. J Proteome Res. 7, 1750–1760 (2008). 18338860 10.1021/pr700878t

[b12] Jung Y. H. . Differential expression of defense/stress-related marker proteins in leaves of a unique rice blast lesion mimic mutant (*blm*). J Proteome Res. 5, 2586–2598 (2006). 17022630 10.1021/pr060092c

[b13] Yin Z. C. . Characterizing rice lesion mimic mutants and identifying a mutant with broad-spectrum resistance to rice blast and bacterial blight. Mol Plant Microbe Interact. 13, 869–876 (2000). 10939258 10.1094/MPMI.2000.13.8.869

[b14] Funayama K. . Cytosolic glutamine synthetase1;2 is responsible for the primary assimilation of ammonium in rice roots. Plant Cell Physiol. 54, 934–943 (2013). 23509111 10.1093/pcp/pct046

[b15] Goodall A. J., Kumar P. & Tobin A. K. Identification and expression analyses of cytosolic glutamine synthetase genes in barley (*Hordeum vulgare L*.). Plant Cell Physiol. 54, 492–505 (2013). 23324171 10.1093/pcp/pct006

[b16] Lam H. M., Coschigano K. T., Oliveira I. C., Melo-Oliveira R. & Coruzzi G. M. The molecular-genetics of nitrogen assimilation into amino acids in higher plants. Annu Rev Plant Physiol Plant Mol Biol. 47, 569–593 (1996). 15012301 10.1146/annurev.arplant.47.1.569

[b17] Forde B. G. & Lea P. J. Glutamate in plants: metabolism, regulation, and signalling. J Exp Bot. 58, 2339–2358 (2007). 17578865 10.1093/jxb/erm121

[b18] Matoh T., Ida S. & Takahashi E. Isolation and characterization of NADH-glutamate synthase from pea (*Pisum sativum L*.). Plant Cell Physiol. 21, 1461–1474 (1980). 25385962 10.1093/pcp/21.8.1461

[b19] Tabuchi M., Abiko T. & Yamaya T. Assimilation of ammonium ions and reutilization of nitrogen in rice (*Oryza sativa L*.). J Exp Bot. 58, 2319–2327 (2007). 17350935 10.1093/jxb/erm016

[b20] Coschigano K. T., Melo-Oliveira R., Lim J. & Coruzzi G. M. *Arabidopsis gls* mutants and distinct Fd-GOGAT genes. Implications for photorespiration and primary nitrogen assimilation. Plant Cell. 10, 741–752 (1998). 9596633 10.1105/tpc.10.5.741 PMC144371

[b21] Jamai A., Salome P. A., Schilling S. H., Weber A. P. & McClung C. R. *Arabidopsis* photorespiratory serine hydroxymethyltransferase activity requires the mitochondrial accumulation of ferredoxin-dependent glutamate synthase. Plant Cell. 21, 595–606 (2009). 19223513 10.1105/tpc.108.063289 PMC2660619

[b22] Ishiyama K., Hayakawa T. & Yamaya T. Expression of NADH-dependent glutamate synthase protein in the epidermis and exodermis of rice roots in response to the supply of ammonium ions. Planta. 204, 288–294 (1998). 9530872 10.1007/s004250050258

[b23] Huang Q. N. . Characterization and genetic analysis of a light- and temperature-sensitive spotted-leaf mutant in rice. J Integr Plant Biol. 53, 671–681 (2011). 21605341 10.1111/j.1744-7909.2011.01056.x

[b24] Park S. Y. . The senescence-induced staygreen protein regulates chlorophyll degradation. Plant Cell. 19, 1649–1664 (2007). 17513504 10.1105/tpc.106.044891 PMC1913741

[b25] Zhou Q. Y. . Knockdown of *GDCH* gene reveals reactive oxygen species-induced leaf senescence in rice. Plant Cell Environ. 36, 1476–1489 (2013). 23421602 10.1111/pce.12078

[b26] Lee R. H., Wang C. H., Huang L. T. & Chen S. C. Leaf senescence in rice plants: cloning and characterization of senescence up-regulated genes. J Exp Bot. 52, 1117–1121 (2001). 11432928 10.1093/jexbot/52.358.1117

[b27] Emanuelsson O., Nielsen H. & von Heijne G. ChloroP, a neural network-based method for predicting chloroplast transit peptides and their cleavage sites. Protein Sci. 8, 978–984 (1999). 10338008 10.1110/ps.8.5.978 PMC2144330

[b28] Emanuelsson O., Nielsen H., Brunak S. & Heijne G. von. Predicting subcellular localization of proteins based on their N-terminal amino acid sequence. J Mol Biol. 300, 1005–1016 (2000). 10891285 10.1006/jmbi.2000.3903

[b29] Suzuki A. & Rothstein S. Structure and regulation of ferredoxin-dependent glutamase synthase from *Arabidopsis thaliana*. Cloning of cDNA expression in different tissues of wild-type and *gltS* mutant strains, and light induction. Eur J Biochem. 243, 708–718 (1997). 9057836 10.1111/j.1432-1033.1997.00708.x

[b30] Sharkey T. D. Estimating the rate of photorespiration in leaves. Physiol Plantarum. 73, 147–152 (1988).

[b31] Noctor G. . Light-dependent modulation of foliar glutathione synthesis and associated amino acid metabolism in poplar overexpressing γ-glutamylcysteine synthetase. Planta. 202, 357–369 (1997).

[b32] Jones D. P. Redefining oxidative stress. Antioxid Redox Signal. 8, 1865–1879 (2006). 16987039 10.1089/ars.2006.8.1865

[b33] Li Z. . Fine mapping of the lesion mimic and early senescence 1 (*lmes1*) in rice (*Oryza sativa*). Plant Physiol Bioch. 80, 300–307 (2014). 10.1016/j.plaphy.2014.03.031 24832615

[b34] Kliebenstein D. J., Dietrich R. A., Martin A. C., Last R. L. & Dangl J. L. LSD1 regulates salicylic acid induction of copper zinc superoxide dismutase in *Arabidopsis thaliana*. Mol Plant Microbe Interact. 12, 1022–1026 (1999). 10550898 10.1094/MPMI.1999.12.11.1022

[b35] Dangl J. L. & Jones J. D. Plant pathogens and integrated defence responses to infection. Nature. 411, 826–833 (2001). 11459065 10.1038/35081161

[b36] Torres M. A., Dangl J. L. & Jones J. D. *Arabidopsis* gp91phox homologues *AtrbohD* and *AtrbohF* are required for accumulation of reactive oxygen intermediates in the plant defense response. Proc Natl Acad Sci USA 99, 517–522 (2002). 11756663 10.1073/pnas.012452499 PMC117592

[b37] Brodersen P. . Knockout of *Arabidopsis accelerated-cell-death11* encoding a sphingosine transfer protein causes activation of programmed cell death and defense. Genes Dev. 16, 490–502 (2002). 11850411 10.1101/gad.218202 PMC155338

[b38] Navabpour S. . Expression of senescence-enhanced genes in response to oxidative stress. J Exp Bot. 54, 2285–2292 (2003). 12947053 10.1093/jxb/erg267

[b39] Sagor G. H. . Reducing cytoplasmic polyamine oxidase activity in *Arabidopsis* increases salt and drought tolerance by reducing reactive oxygen species production and increasing defense gene expression. Front Plant Sci. 7, 00214, 10.3389/fpls 00214 (2016). PMC4770033 26973665

[b40] Voss I., Sunil B., Scheibe R. & Raghavendra A. S. Emerging concept for the role of photorespiration as an important part of abiotic stress response. Plant Biol (Stuttg). 15, 713–722 (2013). 23452019 10.1111/j.1438-8677.2012.00710.x

[b41] Jing S. J., Zhou X., Song Y. & Yu D. Q. Heterologous expression of *OsWRKY23* gene enhances pathogen defense and dark-induced leaf senescence in *Arabidopsis*. Plant Growth Regul. 58, 181–190 (2009).

[b42] Chen H. L. . The Fd-GOGAT1 mutant gene *lc7* confers resistance to *Xanthomonas Oryzae Pv. Oryzae* in rice. Sci Rep. 6, 26411, 10.1038/srep 26411 (2016). 27211925 PMC4876388

[b43] Xie X. Z. . Phytochromes regulate SA and JA signaling pathways in rice and are required for developmentally controlled resistance to *Magnaporthe grisea*. Mol Plant. 4, 688–696 (2011). 21357645 10.1093/mp/ssr005

[b44] Streb P., Shang W., Feierabend J. & Bligny R. Divergent strategies of photoprotection in high-mountain plants. Planta 207, 313–324 (1998).

[b45] Tamura W. . Reverse genetics approach to characterize a function of NADH-glutamate synthase1 in rice plants. Amino Acids. 39, 1003–1012 (2010). 20213442 10.1007/s00726-010-0531-5

[b46] Tamura W. . Disruption of a novel NADH-glutamate synthase2 gene caused marked reduction in spikelet number of rice. Front Plant Sci. 2, 57 (2011). 22645542 10.3389/fpls.2011.00057 PMC3355815

[b47] Hiei Y. & Komari T. Agrobacterium-mediated transformation of rice using immature embryos or calli induced from mature seed. Nat Protoc. 3, 824–834 (2008). 18451790 10.1038/nprot.2008.46

[b48] Wu Z. M. . A chlorophyll-deficient rice mutant with impaired chlorophyllide esterification in chlorophyll biosynthesis. Plant Physiol. 145, 29–40 (2007). 17535821 10.1104/pp.107.100321 PMC1976586

[b49] Wang Z. H. . Functional inactivation of UDP-*N*-acetylglucosamine pyrophosphorylase 1 (UAP1) induces early leaf senescence and defence responses in rice. J Exp Bot. 66, 973–987 (2015). 25399020 10.1093/jxb/eru456 PMC4321554

[b50] Thompson J. D., Gibson T. J. & Higgins D. G. Multiple sequence alignment using ClustalW and ClustalX. Curr Protoc Bioinformatics. Chapter **2**, 2–3 (2002). 10.1002/0471250953.bi0203s00 18792934

[b51] Su N. . Disruption of a rice pentatricopeptide repeat protein causes a seedling-specific albino phenotype and its utilization to enhance seed purity in hybrid rice production. Plant Physiol. 159, 227–238 (2012). 22430843 10.1104/pp.112.195081 PMC3366715

[b52] Chiu W. . Engineered GFP as a vital reporter in plants. Curr Biol. 6, 325–330 (1996). 8805250 10.1016/s0960-9822(02)00483-9

[b53] Wang Y. C., Zhang Y., Wang Z., Zhang X. Y. & Yang S. H. A missense mutation in CHS1, a TIR-NB protein, induces chilling sensitivity in *Arabidopsis*. Plant J. 75, 553–565 (2013). 23651299 10.1111/tpj.12232

[b54] Manosalva P. M., Bruce M. & Leach J. E. Rice 14-3-3 protein (GF14e) negatively affects cell death and disease resistance. Plant J. 68, 777–787 (2011). 21793954 10.1111/j.1365-313X.2011.04728.x

