# Supplementary material for: Isolation and characterization of a spotted leaf 32 mutant with early leaf senescence and enhanced defense response in rice
Source: Sci Rep. 2017 Jan 31;7:41846. doi: 10.1038/srep41846 (PMC5282590; doi:10.1038/srep41846)
Supplement: Supplementary Figures and Tables [file srep41846-s1.pdf]

Supplemental information

Title: *SPL32*, encoding a Fd-GOGAT, plays an important role in leaf senescence and defense response in rice (*Oryza sativa* L)

Liting Sun[1,*], Yihua Wang[1*], Ling-long Liu[1], Chunming Wang[1], Ting Gan[1], Zhengyao Zhang[1], Yunlong Wang[1], Di Wang[1], Mei Niu[1], Wuhua Long[1], Xiaohui Li[1], Ming Zheng[1], Ling Jiang[1] & Jianmin Wan[1,2]

[1]State Key Laboratory for Crop Genetics and Germplasm Enhancement, Jiangsu Plant Gene Engineering Research Center，Nanjing Agricultural University, Nanjing 210095, China. [2]National Key Facility for Crop Gene Resources and Genetic Improvement, Institute of Crop Science, Chinese Academy of Agricultural Sciences, Beijing 100081, China. *These author contribute equally to this work. Correspondence and requests for materials should be addressed to Jianmin Wan (E-mail: wanjm@njau.edu.cn or wanjianmin@caas.cn; Tel & Fax: +86-25-84396516).

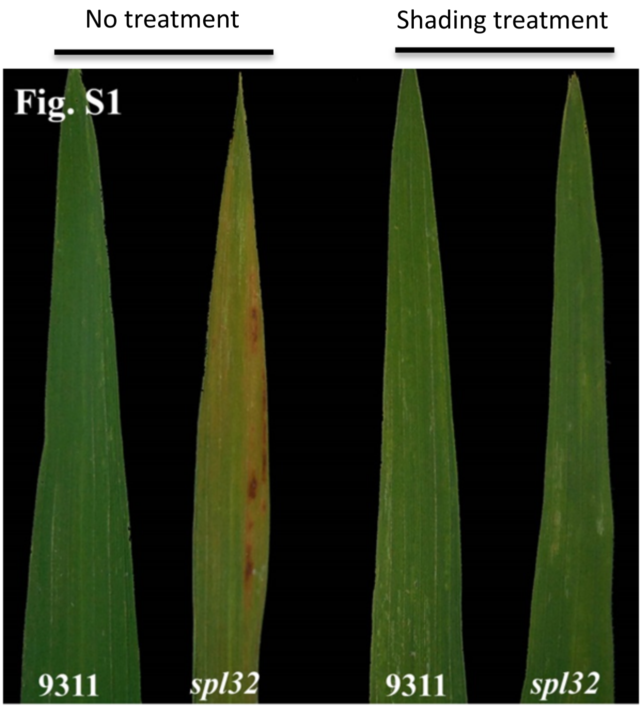

Supplemental Figure 1. Light induced experiment. We chose the leaves in the tillering stage. Leaves of 9311 and *spl32* mutant on the right panel were covered by tinfoil for 10 days.

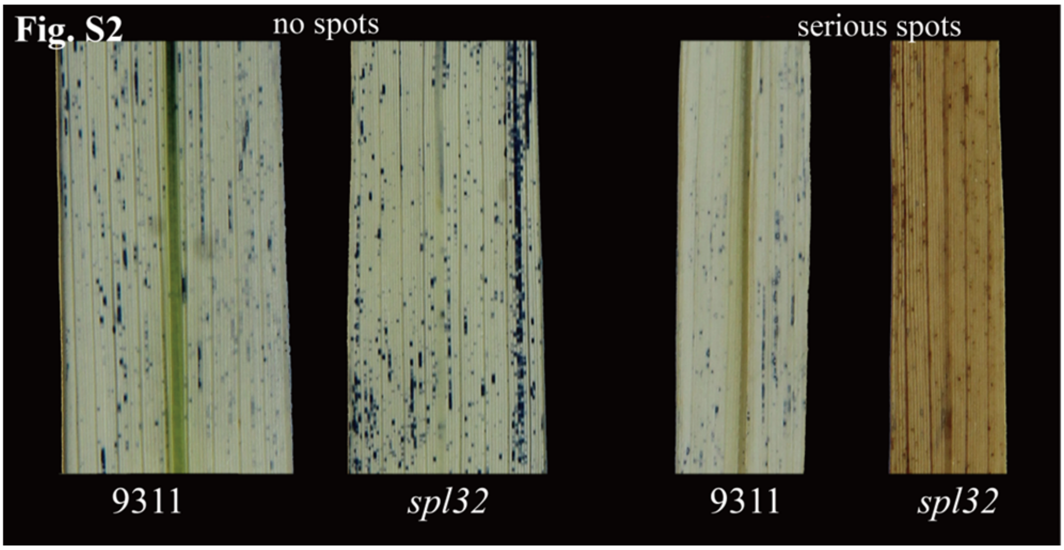

Supplemental Figure 2. Dying experiments. Leaves with no spots or serious spots from *spl32* mutant and the corresponding sections of leaves in the wild-type 9311 at tillering stage were stained by nitrotetrazolium blue chloride

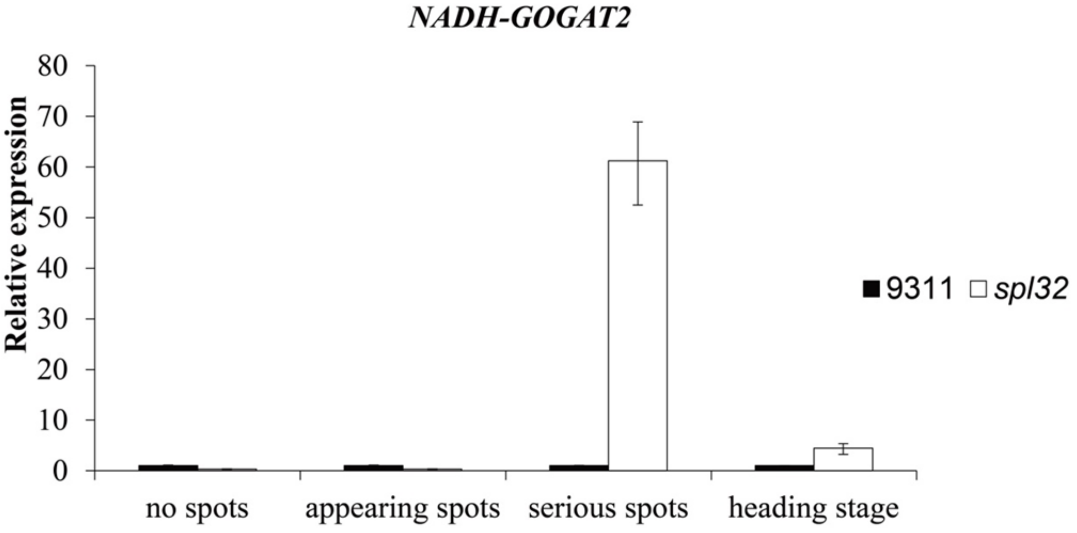

Supplemental Figure3. Expression of *NADH-GOGAT2* in leaves with or without lesion spots at different developmental stages.

Supplemental Table S1 Segregation of the F_2 population from the wild-type 9311 and *spl32* mutant

Cross	WT	Mutant	The actual separation ratio	$\chi^2_{(3:1)}(\chi^2_{0.05}<3.84)$
spl32 × 9311	117	38	3.08:1	0.0064
9311 × *spl32*	101	35	2.89:1	0.0294

Supplemental Table S2 Polymorphic InDel markers between parents in target region

primer	Forward primer sequence	Reverse primer sequence	PAC number
zzy-2	TTAGCCAATTCCATCAAG	ATTTCTTTCCAGCGACAA	OJ1458_B07
zzy-3	CATTTGACCGTTCATCTT	TGACATCTCAGCGTTGCA	P0470D12
zzy-18	ATTGGTGGGTTGCTTGGT	GTGGGCATAGCCTTTGGT	OJ1477_F01
zzy-20	GGATCAATGCCATCTGTT	GCAATAAGCACTTGTTTCG	OJ1477_F01
zzy-21	GACAGACAACTTGGGAACA	GTTTGCAGTCTTGACTCTTG	OJ1477_F01
zzy-22	TATCCCTTTCCTGTTCGT	TTGGAAAGACACCAGAAA	OJ1477_F01
zzy-23	AGACAGAGCCTTATTCAA	AACCTCCCAACCTTTCTA	P0496C02
zzy-24	CGTTACTTTGTTCGTCCTA	CTTGTCACGCATACTGTTG	P0496C02
zzy-30	ATGGATGGTTAACGTTCT	CTTAGCACTGGCTCTTCA	P0047B07
zzy31	TTGCATGATATTCACCTG	CCTATAAGATTCCCTAAAGT	OJ1477_F01

Supplemental Table S3 Gene-specific primers used in this study

Primers	Oligos	Function
Fd-GOGAT-GFP-F	AGCCCAGATCAACTAGTATGGCCACGCTCCCACGTG	Subcellular
Fd-GOGAT-GFP-R	CACCATGGATCCCCCGGGCTTCGCCGATTGTACTGTTGT	Localization
CWW-11-1-F	TGGGTGGTGGTAGACGAG	Cloning of
CWW-11-1-R	CACTTGGGAATTGCAGTAGA	*Fd-GOGAT*
zf-1-F	TGACTGGGCCAACAAGCAA	Verification of
zf-1-R	CATAGCGACCTACCACTGATACATT	57-bp deletion
		in *spl32*
GT-2 F	TGCAGGCCAGCTCAACATTACG	qRT-PCR for
GT-2 R	TCTCCACCAGCCATACCCTTTC	expression
		pattern analyses
Flag-F	TTCATTTCATTTGGAGAGAA	Complemented
Flag-R	GGTACCCCGGGTTCGAAATC	verification
NADH-2 F	GGAAGATACGGACCAACT	qRT-PCR
NADH-2 R	AACATACAGAAGCAGCATT	
PR1a F	TTCATCACCTGCAACTACTCG	qRT-PCR
PR1a R	TGCATAAACACGTAGCATAGCAT	
PR1b F	GTGTGCGGGCACTACACG	qRT-PCR
PR1b R	CGGCTTATAGTTGCATGTGA	
PR5 F	ATCGACGGCTACAACGTC	qRT-PCR
PR5 R	GTGTCTTGGTGTTGTCTTCG	
PR10 F	CACCATCTACACCATGAAGC	qRT-PCR
PR10 R	AGCACATCCGACTTTAGGAC	
